# Speech Development Between 30 and 119 Months in Typical Children II: Articulation Rate Growth Curves

**DOI:** 10.1044/2021_JSLHR-21-00206

**Published:** 2021-09-29

**Authors:** Tristan J. Mahr, Jennifer U. Soriano, Paul J. Rathouz, Katherine C. Hustad

**Affiliations:** a Department of Communication Sciences and Disorders, University of Wisconsin–Madison; b Waisman Center, University of Wisconsin–Madison; c Department of Population Health, Dell Medical School, The University of Texas at Austin

## Abstract

**Purpose:**

We aimed to develop normative growth curves for articulation rate during sentence repetition for typically developing children. Our primary goal was the development of quantile/percentile growth curves so that typical variation in articulation rate with age could be estimated. We also estimated when children became adultlike in their articulation rate, and we examined the contributions of age and utterance length to articulation rate.

**Method:**

This cross-sectional study involved collection of in-person speech samples from 570 typically developing children (297 girls; 273 boys) who passed speech, language, and hearing screening measures. Pauses greater than 150 ms in duration were removed from the samples, and articulation rate was measured in syllables per second (sps).

**Results:**

Articulation rate reliably increased with age and utterance length. Rate in all key percentiles increased with age. The median rate (50th percentile) increased from 2.7 sps at 36 months to 3.3 sps at 96 months. The 5th percentile increased from 2.3 to 3.1 sps over the same age range. Using 3.2 sps as a benchmark for adultlike speech, we found the 25th, 50th, and 75th percentiles reached adultlike rates at 99, 75, and 53 months, respectively.

**Conclusions:**

Articulation rate increases from early childhood into middle childhood, and it is generally adultlike by 10 years of age. Variability in articulation rate among typical children was substantial. Implications for prior research and for clinical usage are discussed.

Speech production requires the complex coordination of respiratory, phonatory, resonatory, and articulatory speech subsystems as well as the overlaid features of prosody (rate, stress, and intonation), all demanding rapid sequential movement through space and time. Acquisition of mature speech production abilities is a complex protracted process that occurs over the course of years and involves considerable anatomical growth, refinement of speech motor control, and cognitive and language development. Many aspects of speech development in children have received significant research attention both recently and historically, including anatomical growth (e.g., Vorperian et al., 1999, 2005, 2009), speech motor control development (e.g., Namasivayam et al., 2020; Smith & Goffman, 1998; Smith & Zelaznik, 2004; Vick et al., 2012), speech acoustics (e.g., Hillenbrand et al., 1995; Lee et al., 1999; Vorperian & Kent, 2007), respiratory development (e.g., Boliek et al., 1997; Connaghan et al., 2004; Hoit et al., 1990), speech sound development (e.g., Crowe & McLeod, 2020; McLeod & Crowe, 2018; Sander, 1972; Smit et al., 1990), and most recently speech intelligibility development (e.g., Hustad et al., 2020, 2021). The study of speech rate development has also received attention (e.g., Haselager et al., 1991; Nip & Green, 2013; Tendera et al., 2019), but the developmental picture of how speech rate—and in particular, articulation rate—advances over time remains unclear.

This article is the second in a series examining developmental change in speech production for typical children between preschool and middle childhood. In the first paper (Hustad et al., 2021), we examine speech intelligibility development for single words and connected speech for a large cross-sectional group of children, generating percentile growth curves and quantifying the effects of utterance length on intelligibility by age. In the present article, we examine parallel data on the developmental change in articulation rate, creating percentile growth curves and quantifying the effects of utterance length on articulation rate. In a subsequent paper, we will present integrated findings of the impacts of speech rate on intelligibility for the same data set. The end goal of this work is to establish normative references for intelligibility and rate that can be used as benchmarks for identifying children who are delayed or disordered. This descriptive and developmental work is a particular need in pediatric dysarthria, where features of typical development overlap considerably with features of speech motor impairment (Schölderle et al., 2020).

As noted, our primary interest for the present article is development of *articulation rate,* which is differentiated from *speech rate.* As a more global measure of speech tempo, *speech rate* describes the overall rate of production during speech and includes two components: duration of speech and duration of pauses. In contrast, articulation rate *excludes* all pause time, with pauses generally defined as silent intervals with a minimum duration of 150–250 ms (e.g., 150 ms for Redford, 2014; 250 ms for Walker et al., 1992). Common measurement units for both speech rate and articulation rate include phones per second or syllables per second (sps). To clarify the distinction, we might think of articulation rate as timing the production of *articulatory movements for speech*—where pauses require the cessation of these movements. Speaking rate and articulation rate often coincide, especially for short or elicited speech samples where there are no pauses; one can articulate relatively quickly yet speak relatively slowly by manipulating pause behaviors. Goldman-Eisler (1956) measured speech output in adult interviews and found that speaking rate was more strongly correlated with the proportion of pause time (*r* = −.94) than with articulation rate (*r* = −.17, *ns.*). Because pause behavior can influence speaking rate, articulation rate provides a more direct measure of the efficiency of the articulators during speech than speaking rate. Tsao and Weismer (1997), for example, suggest that articulation rate may be a reflection of neuromotor constraints.

Research on the development of speech and/or articulation rate has varied widely with respect to age range and number of children studied, speaking tasks, research design (cross-sectional vs. longitudinal), and findings. Collectively, results indicate some overlap of findings among studies and some inconsistent findings among studies. For instance, some studies have shown unclear or inconsistent age differences (Pindzola et al., 1989; Sturm & Seery, 2007; Walker & Archibald, 2006). Others have found clear growth between ages (Nip & Green, 2013; Walker et al., 1992). A key conclusion across studies is that articulation rate does increase with age. Furthermore, there seems to be a differential effect of utterance length on articulation rate, at least for older children (Darling-White & Banks, 2021). However, the magnitude of rate change over time is less clear from existing studies. Redford (2014) highlights that a main theme in the literature is variability among individuals, even when speech material is controlled. Most studies to date have employed small numbers of children at any given age and thus are underpowered considering the variability among children. Large-scale studies are needed to advance our understanding of the time course of rate development in children and to characterize the variability in this time course.

Previous studies have suggested that articulation rate is an important predictor of speech motor deficits (i.e., dysarthria) in children. For example, Allison and Hustad (2018a) compared various acoustic features on how well they classified 5-year-old children with dysarthria (secondary to cerebral palsy) versus those with typical development. Articulation rate was a robust predictor of dysarthria status, and 13 of 20 children with dysarthria had an articulation rate that fell below the typically developing group mean by 2 *SD*s. In addition, Allison and Hustad (2018b) found that articulation rate was a key differentiating variable between subgroups of children with cerebral palsy and dysarthria who had equivalent levels of intelligibility. Collectively, these findings suggest that articulation rate is a sensitive measure for detecting speech motor differences in children.

The ability to quantitatively identify children falling below age expectations on articulation rate would be an important clinical advancement, especially for children who are at risk for speech motor deficits, such as those who have cerebral palsy or other developmental speech disorders. Often, children present with subtle deficits that are difficult to differentiate from the range of variability associated with typical development. In many cases, these mild or borderline children may be the ones who are particularly responsive to early speech therapy that may serve to prevent a developmental gap from widening with age.

For articulation rate to be a useful clinical index for children, a comprehensive understanding of the development of rate and the ways that it varies among typical children is essential. Such information would lay the foundation for establishing benchmarks and cut points for the identification of children with typical speech development and those falling outside of that range. There is a critical need for large-scale studies examining development of both articulation rate and speech rate. A key goal of this study was to establish a set of normative references for development of articulation rate using consistent and controlled speech material, employing methods that could be replicated in clinical settings and could be useful in differentiating children with typical and atypical rate development. We examine articulation rate because it enables direct inferences about the development of speech movement without the confound of pause behavior, and it is a necessary prerequisite for interpreting the integration of pause behavior with speech movement in subsequent studies.

In this study, our primary goal was to develop growth curves and identify developmental percentiles based on measures of articulation rate for typical children between the ages of 30 and 119 months (2;6 to 9;11 [years;months]). We also present data showing the impact of utterance length on articulation rate by age to identify whether the finding of increased articulation rate with longer utterances observed by Darling-White and Banks (2021) holds true at earlier ages. Our second goal was to determine the age at which children become adultlike in their articulation rate and to examine the extent to which children in different developmental percentiles vary in their age of reaching adultlike articulation rate. To accomplish these goals, we studied the same sample as used in Hustad et al. (2021). Research objectives were as follows:

1. Describe the growth in articulation rate between 30 and 119 months of age as a function of age and utterance length.
2. Quantify growth in speech rate between 30 and 119 months of age in typically developing children and characterize the range of typical development by age in terms of percentiles.
3. Estimate the distribution of ages at which typically developing children become adultlike in their speech rate development.

The primary goal for this work was to develop percentile growth curves for articulation rate as in Objective 2. Objective 1 performed groundwork for the growth curves by describing and visualizing the observed articulation rates by age and utterance length. Darling-White and Banks (2021) and Redford (2014) both found increases in articulation rate with utterance length, so it was important to examine age effects with various utterance lengths. For Objective 3, we used the 10th percentile of articulation rate for adults as a baseline for defining adult performance and estimated when different percentile curves crossed this baseline. Because previous studies have yielded conflicting results, our hypotheses were a set of general developmental expectations. We predicted that the average articulation rate of children would increase with age as their speech motor systems matured and they become more practiced speakers. We also expected variability in articulation to decrease with age as children converge onto an adult level of variability.

## Method

This study was reviewed and approved by the University of Wisconsin–Madison Institutional Review Board (Social and Behavioral Sciences). We obtained informed consent from or on behalf of all participants. Data collection methods in this article are the same as those employed in Hustad et al. (2020); thus, we provide abbreviated descriptions here. Note that this is a companion paper to Hustad et al. (2021), which reports data on development of speech intelligibility for typically developing children.

### Participants

*Typically developing children*. We recruited a community sample of typically developing children through public postings, including flyers posted in local venues, and online advertisements. We also accessed a research registry that enrolls children from local public schools, maintained by the Waisman Center Clinical Translational Core for recruitment. Inclusion criteria were (a) age between 30 and 119 months (2;6–9;11), (b) American English as the primary language in the home, (c) hearing within normal limits as indicated by pure-tone hearing screening or distortion product otoacoustic emission screening bilaterally or by parent report for very young children who did not tolerate screening, (d) speech within normal limits as indicated by scores from standardized articulation testing (Fudala, 2001), and (e) language within normal limits as indicated by scores from standardized language testing (Wiig et al., 2013; Zimmerman et al., 2012). Children receiving intervention services for any educational or developmental concern were excluded along with those having any medical diagnoses related to development.

The companion study focusing on speech intelligibility development (Hustad et al., 2021) examined a community-based sample of 538 children (282 girls and 256 boys). This study included an additional 32 children (15 girls and 17 boys) for whom we were not able to gather intelligibility data due to the COVID-19 pandemic. The total number of children included in this study was 570 children (273 boys and 297 girls). See Hustad et al. (2021) for details regarding children who were excluded from this research.

All children were from the upper midwestern region of the United States, and their demographic characteristics reflect those of Wisconsin. Table 1 shows demographic information for the children. Table 2 shows the age and sex distribution of speakers.

**Table 1.** Demographic characteristics of children (*N = 570*).

Characteristic	Male (*n* = 273)	Female (*n* = 297)
Race		
White	242 [11]	250 [6]
Black	10 [1]	2
Asian	3	6
American Indian		1
Native Hawaiian/Pacific Islander		1
More than 1 race	11 [1]	26 [2]
Other		
Not reported	7	11
2-Factor Hollingshead Social Index mean	55.45 (8.09)	55.50 (8.00)
Maternal education		
Graduate degree or graduate professional training	126	142
Standard college or university degree	125	116
Partial college or specialized training	11	23
High school graduate	6	4
Not reported	5	12

*Note.* Note that all children who were included in this study passed speech, language, and hearing screening measures and, thus, were all within normal age-level parameters. The numbers of additional children in this racial category whose parents identified them as having Hispanic ethnicity are indicated in []. All other children were identified as non-Hispanic. Standard deviations are indicated by (). Hollingshead Social Index is a composite measure of socioeconomic status.

**Table 2.** Age and sex distribution of typically developing children and adult speakers.

Age range	*n* children	*n* boys	*n* girls	Mean age
30–35	57	28	29	33
36–41	50	24	26	38
42–47	58	20	38	45
48–53	53	18	35	50
54–59	59	31	28	57
60–65	51	28	23	63
66–71	52	28	24	68
72–77	44	24	20	75
78–83	57	27	30	80
84–89	27	13	14	86
90–95	19	8	11	92
96–101	10	4	6	99
102–107	13	4	9	105
108–113	8	7	1	110
114–119	12	9	3	116
Adults				
216–317 (18;0–26;5 [years;months])	23	12	11	257 (21;5)

*Note.* Note that younger children were oversampled because of the variability demonstrated in our earlier study (Hustad et al., 2020). Age is reported in months.

*Adult speakers*. We also recruited a community sample of adult speakers to serve as a comparison group or as a reference point of the endpoint of development. Recruitment occurred via campus postings. Inclusion criteria were (a) hearing within normal limits as indicated by pure-tone hearing screening; (b) age between 18 and 45 years; (c) American English as their first language; and (d) no identified language, learning, or cognitive disabilities per self-report. In total, 23 adults (12 men and 11 women) contributed speech samples for this study. The mean age of adult speakers was 21;5 (*SD* = 2;1).

### Materials and Procedures

#### Acquisition of Speech Samples From Children

Detailed information regarding procedures for collecting speech samples from children is provided in our companion paper (Hustad et al., 2021). Briefly, for all children in this study, we elicited the same standard set of utterances taken from the Test of Children's Speech (Hodge & Daniels, 2007). Child productions were elicited for 38 single words and up to 60 sentences; our rate measurements only used samples from the sentences. Sentences systematically ranged in length from two to seven words, with 10 utterances of each length.^1^ Children heard a recorded model of target utterances along with a picture illustrating the content and were asked to produce each utterance after they heard it. Note that we also examined articulation rate for the elicitation recordings presented to children; these data are reported for Objective 3. Children's speech was recorded in person using a professional-quality digital audio recorder (Marantz PMD 570) at a 44.1-kHz sampling rate (16-bit quantization) and a condenser studio microphone (Audio-Technica AT4040) positioned 18 in. from the child's mouth. Recordings were monitored in real time and recording levels were adjusted on a mixer (Mackie 1202 VLZ) to obtain optimized recordings.

#### Determining Articulation Rate

In this study, our outcome variable of interest was articulation rate. Articulation rate quantifies how quickly the articulators are moving to produce speech units (in this case, syllables) per unit of time (in this case, seconds), excluding pauses that are greater than 150 ms. The main analytic tasks for calculating articulation rate were counting the number of syllables and computing the time spent speaking and the time spent pausing. All three of these tasks were completed as part of a semisupervised forced alignment procedure.

*Forced alignment*. After speech samples were recorded, a child's productions were segmented into individual utterances (i.e., individual .wav files). Lexical transcripts of the words produced for each utterance (and paired .wav file) were then created by research assistants for each child. We then segmented the recordings of individual utterances with their associated transcripts using the Montreal Forced Aligner (version 1.01; McAuliffe et al., 2017) with speaker adaptive training enabled. We used this aligner based on our previous evaluation of aligners for children's speech (Mahr et al., 2021). Briefly described, *forced alignment* uses a statistical acoustic model and a speech transcript to segment an audio recording into portions of time for individual words and the phones in those words. The phones are retrieved from a pronunciation dictionary, and silence is treated as a special pseudoword/pseudophone; thus, silence can be segmented as well. The output of forced alignment is a Praat textgrid (version 6.1; Boersma & Weenink, 2021) with a tier for individual words and a tier for individual phones in those words. Syllable counts for speech rate measurements were obtained from the pronunciation dictionary transcriptions.

*Pause identification*. To measure articulation rate, we excluded all pauses that were 150 ms or longer in duration. We chose 150 ms based on prior work on pauses (e.g., Redford, 2014). We also excluded silence at the beginning and end of the alignment (i.e., before and after the utterance). We used a semisupervised approach for pause identification, relying on forced alignment to identify pause candidates but manually reviewing and correcting those candidates. Any file with 100 ms of silence between segments of speech (i.e., pauses and “near” pauses) was marked for review, as were files with 1,000 ms of silence at the beginning or end of the file (i.e., possible forced alignment errors). When identifying pause intervals, we did not count the neighboring 50 ms of silence preceding or following a stop consonant (or preceding an affricate) to account for closure of the articulators (Crystal & House, 1988). Of the 30,940 recordings, 5,183 met one of these review criteria. Research assistants reviewed these files and used Praat to manually correct the textgrid boundaries for intervals of silence. Following review, we computed articulation rate as the number of syllables per second of speech duration after excluding pauses.

*Imputation and weighting*. Not all children were able to repeat longer length utterances. For example, only two of 57 children in the 30- to 35-month age range were able to repeat five-word utterances. To compute developmental growth curves, we calculated an overall articulation rate by imputing the rates for unobserved utterance lengths and taking an age-based weighted average of the articulation rates for each utterance length. We describe the procedure in detail in Hustad et al. (2020), but we summarize the two main steps here.

First, we calculated the imputed articulation rate as the imputed number of syllables divided by the imputed duration. We fit linear models on the observed data that estimated the value for a variable (syllables or duration) at length *L* as a linear combination of the variable for all shorter utterance lengths *l* < *L* and the length of longest utterance attained by the child. We used successive predictions from these models to impute missing values. Thus, for a child missing a value in five-word utterances, we imputed the five-word value from the child's length of longest utterance length (four words) and the value in two-, three-, and four-word utterances. This five-word value was then used as a predictor for the six-word prediction. At the end of the process, every child had six articulation rates, one for each utterance length.

Second, we computed overall articulation rate as a weighted average of the rate for each utterance length. The weight for length *L* was based on the probability of reaching length *L* at a given age. For instance, because no children under 35 months of age reached six- or seven-word utterances, the imputed rates for six- and seven-word utterances were down-weighted to nearly 0. After 6 years of age, the utterances lengths were all equally weighted to 1/6.

### Analysis

We report results first with unweighted, observed articulation rates to examine basic length and age effects (Objective 1) and use the imputed and weighted rates for percentile growth curves (Objectives 2 and 3).

For Objective 1, we used conventional linear regression models when there were not repeated measurements (estimating rate as a function of age with a separate model for each utterance length). We used linear mixed-effects models whenever the data featured repeated measurements, as in our models where we regressed articulation rate on utterance length for different age bins (where a speaker had articulation rates from different utterance lengths).

For Objectives 2 and 3, we used a generalized gamma regression model to estimate articulation rate as a flexible function of age. The gamma distribution can be used to model continuous positive values such as rates or average waiting times. The generalized gamma distribution used in this analysis is governed by three parameters—location (mean), scale (variability), and shape (skewness)—each of which we modeled as a function of age. To allow for model flexibility, we modeled age using natural cubic splines. The location parameter was modeled with a 3-degree-of-freedom (*df*) spline, the scale parameter with a 2-*df* spline, and the shape parameter with a 1-*df* (linear) term.

From the fitted model, we computed percentile growth curves. For example, for a given age, say 48 months, we can obtain the predicted values for the location (3.0 sps), scale (0.1), and shape (1.8) for the fitted gamma distribution at age 4;0. Given these parameter estimates, we can use the distribution's quantile function to get estimated speech rates for the 5th (2.5 sps), 10th (2.6), and other percentiles. Figure 3 shows percentile growth curves estimated from our analysis, and Table 4 provides a numerical summary of percentiles by age for reference.

To quantify the uncertainty from model estimates, we used the model's parameter estimates and the estimated robust variance–covariance matrix, and we sampled 10,000 new parameter values from a multivariate normal distribution. For each draw of parameter values, we estimated a new set of percentile growth curves. Taking the .025 and .975 quantiles on the sampled growth curves provided a 95% interval on model estimates. In our earlier example, our estimate for the 5th percentile at age 4;0 would have a 95% interval of [2.4, 2.5] sps.

We carried out this analysis in the R programming language (version 4.0.4; R Core Team, 2021) using the gamlss package (version 5.3.2; Rigby & Stasinopoulos, 2005). Mixed models for Objective 1 were fitted using lme4 (version 1.1.26; Bates et al., 2015).

## Results

### Describe the Growth in Articulation Rate Between 30 and 119 Months of Age as a Function of Age and Utterance Length

**Figure 1.**
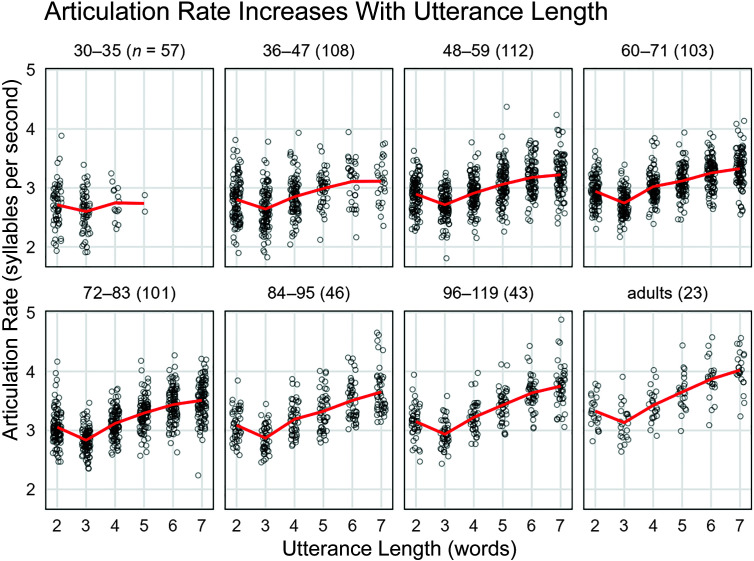
Effects of utterance length on articulation rate (in syllables per second). Panels group speakers into age bins with age reported in months. Within each panel, the figure shows 1 point per child per utterance length provided that the child was able to produce utterances of that length. Lines connect the mean articulation in each utterance length. There was a noticeable decrease in rate from two-word to three-word utterances, but from three words to longer lengths, the mean articulation rate increases with utterance length. As in Table 3, we collapse 8- and 9-year-olds together because of the sparser sampling in this age range.

To examine the effect of utterance length on articulation rate, we assigned children into seven age groups. Figure 1 shows the average articulation rate as a function of age group (facets) and utterance length (*x*-axis). Note that we combined 8- and 9-year-olds into a single group because of the smaller number of participants in this range. Although there was an appreciable dip in articulation rate for two-word versus three-word utterances, the overall trend showed the average articulation rate increasing with utterance length. We confirmed this trend by fitting a mixed-effects regression model for each age group. We regressed articulation rate measured in sps on utterance length, coded as a simple integer value to describe the change in articulation rate for a one-word change in length, and included child-level random slopes so that children could vary in their average articulation rates and their utterance length effects. The models showed a significant effect of utterance length on articulation rate for all groups except for the 30- to 35-month age group, *b*_30–35_ = −0.04 sps per word, 95% CI [−0.09, 0.01], *p* = .10; these children lacked data for longer utterance lengths. In the groups of children with significant utterance length effects, the effect was smallest for the 36- to 47-month group, *b*_36–47_ = 0.07 sps per word, [0.05, 0.09], *p* < .001; and greatest for the 96- to 119-month group, *b*_96–119_ = 0.15 sps per word, [0.14, 0.17], *p* < .001. For the adult speakers, the effect was even greater, *b*_adults_ = 0.17 sps per word, [0.15, 0.19], *p* < .001.

**Figure 2.**
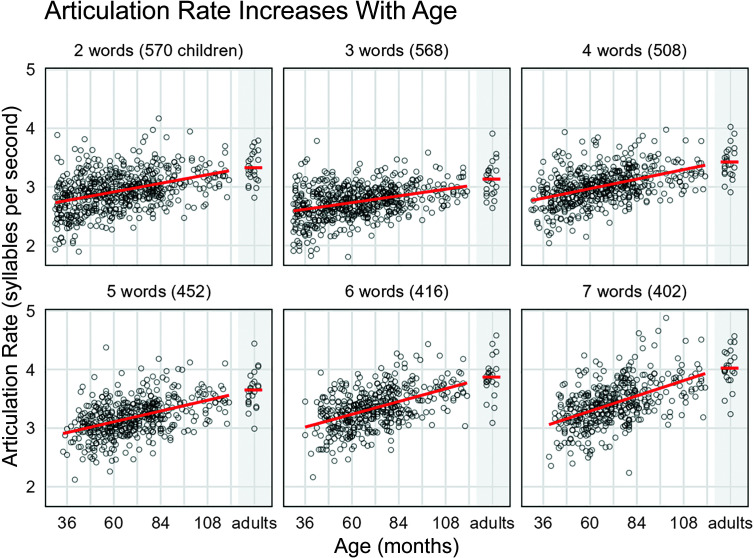
Effects of age on articulation rate (in syllables per second). Within each panel, the figure shows 1 point per speaker, provided that the child was able to produce utterances of that length. Rates from 23 adult speakers are included. Long line shows a linear regression fitted separately for each panel, and short line shows the adult-age mean rate. Articulation rate increased with age for all utterance lengths, and the slope of this relationship increased with utterance length.

Figure 2 shows the effect of age within each utterance length, and Table 3 provides a numerical summary of these data. For each utterance length, we fit a linear model of articulation rate as a function of age in months divided by 12 so that coefficients describe the expected change in sps for a 1-year change in age. There was significant effect of age for every utterance length, with the smallest effect for three-word utterances, *b*_3-word_ = 0.06 sps per year, 95% CI = [0.04, 0.07], *p* < .001; and the largest effect for seven-word utterances, *b*_7-word_ = 0.13 sps per year, [0.11, 0.15], *p* < .001.

**Table 3.** Mean articulation rate in syllables per second in various age bins.

Age range (months)	Overall			2 words			3 words			4 words			5 words			6 words			7 words		
	*n*	*M*	*SD*	*n*	*M*	*SD*	*n*	*M*	*SD*	*n*	*M*	*SD*	*n*	*M*	*SD*	*n*	*M*	*SD*	*n*	*M*	*SD*
30–35	57	2.7	0.32	57	2.7	0.37	56	2.6	0.34	14	2.7	0.29	2	2.7	0.20	—	—	—	—	—	—
36–47	108	2.8	0.34	108	2.8	0.37	107	2.6	0.34	89	2.8	0.34	46	3.0	0.29	31	3.1	0.39	25	3.1	0.37
48–59	112	3.0	0.27	112	2.9	0.28	112	2.7	0.24	112	2.9	0.27	111	3.1	0.32	93	3.2	0.30	87	3.2	0.34
60–71	103	3.1	0.23	103	2.9	0.25	103	2.7	0.22	103	3.0	0.25	103	3.1	0.25	102	3.3	0.26	100	3.3	0.30
72–83	101	3.3	0.24	101	3.1	0.30	101	2.8	0.21	101	3.1	0.24	101	3.3	0.27	101	3.4	0.29	101	3.5	0.33
84–95	46	3.3	0.30	46	3.1	0.27	46	2.9	0.23	46	3.2	0.28	46	3.3	0.33	46	3.5	0.33	46	3.7	0.38
96–119	43	3.4	0.26	43	3.1	0.29	43	2.9	0.23	43	3.2	0.25	43	3.4	0.26	43	3.6	0.28	43	3.7	0.36
Adults	23	3.6	0.28	23	3.3	0.26	23	3.1	0.30	23	3.4	0.27	23	3.7	0.31	23	3.9	0.34	23	4.0	0.32

*Note.* The overall rate is the rate value following for the imputation and weighting procedure used to average across utterance lengths. These values were used in the growth curve models. The rates for each of the utterance lengths (from 2 to 7 words) are the observed rates. These values are plotted in Figures 1 and 2. As in Figure 1, we collapse 8- and 9-year-olds together because of the sparser sampling in this age range. Em dashes indicate that no data exist for these cells.

We note that the model coefficients reported for the simple effects of age and for utterance length above have the same magnitude: The effect of increasing an utterance length by one word is similar in effect size to increasing the speaker's age by 1 year, increasing articulation rate by 0.05–0.15 sps.

Given the main effects of age and utterance length, we augmented the mixed-effects model for utterance length to include an age-by-utterance length interaction. This interaction was statistically significant, *b* = 0.02, 95% CI = [0.01, 0.02], *p* < .001, such that the effect of utterance length on articulation rate increased with age. At 36 months of age, a one-word increase in utterance length provides an expected increase in articulation rate of 0.06 sps, 95% CI [0.05, 0.07], and this estimated effect is larger at 60 months, *b* = 0.10 sps per word, [0.09, 0.10], and still larger at 84 months, *b* = 0.13 sps per word, [0.12, 0.13].

### Quantify Growth in Speech Rate Between 30 and 119 Months of Age in Typically Developing Children and Characterize the Range of Typical Development by Age in Terms of Percentiles

**Figure 3.**
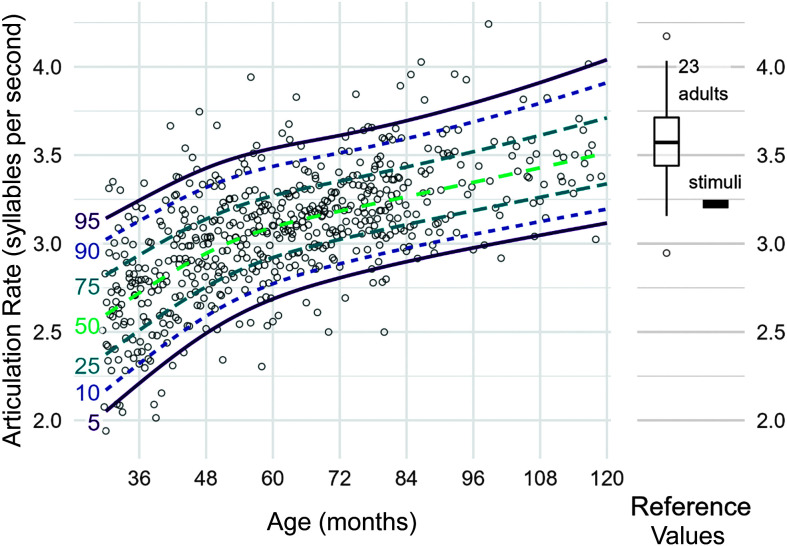
Percentile growth curves for articulation rate (in syllables per second) based on generalized gamma regression models. Points represent the rates from 570 typically developing children. The right panel includes reference values including the mean rate of the stimuli that children heard and a box plot for the average articulation rate for 23 adults. The box of the box plot shows the 25th, 50th, and 75th percentiles.

Figure 3 shows the estimated percentile growth curves for articulation rate for the 5th, 10th, 50th, 90th, and 95th percentiles of children, and Table 4 reports these values numerically. At age 36 months, the estimated articulation rate for the 50th percentile is 2.7 sps, 95% CI [2.7, 2.8]. This value increases to 3.3 sps, [3.3, 3.4], at 96 months. Over the course of 5 years, the median articulation rate on this task increased by 0.63 sps, [0.55, 0.70]. Higher percentiles showed less growth, and lower percentiles showed more growth. The 90th percentile started with a relatively higher articulation rate, so it showed a comparatively smaller 5-year change in estimated articulation rate of 0.56 sps, [0.45, 0.67], from 3.1, [3.1, 3.2], to 3.7 sps, [3.6, 3.8]. For comparison, the 10th percentile showed a larger 5-year change of 0.73 sps, [0.65, 0.82], from 2.3, [2.3, 2.4], to 3.1 sps, [3.0, 3.1].

**Table 4.** Model-estimated articulation rates (syllables per second) by percentile and age.

Age	5th	10th	25th	50th	75th	90th	95th
30	2.1	2.2	2.4	2.6	2.8	3.0	3.1
36	2.2	2.3	2.5	2.7	2.9	3.1	3.2
42	2.4	2.5	2.6	2.8	3.0	3.2	3.3
48	2.5	2.6	2.8	2.9	3.1	3.3	3.4
54	2.6	2.7	2.8	3.0	3.2	3.4	3.5
60	2.7	2.8	2.9	3.1	3.3	3.4	3.5
66	2.8	2.8	3.0	3.1	3.3	3.5	3.6
72	2.8	2.9	3.0	3.2	3.4	3.5	3.6
78	2.9	2.9	3.1	3.2	3.4	3.6	3.7
84	2.9	3.0	3.1	3.3	3.4	3.6	3.7
90	2.9	3.0	3.1	3.3	3.5	3.6	3.7
96	3.0	3.1	3.2	3.3	3.5	3.7	3.8
102	3.0	3.1	3.2	3.4	3.6	3.7	3.9
108	3.0	3.1	3.3	3.4	3.6	3.8	3.9
112	3.1	3.2	3.3	3.5	3.7	3.9	4.0

*Note. N* = 570 typically developing children. Age reported in months.

There was also a relatively consistent amount of variability among children. At 36 months, the estimated difference between the 95th and 5th percentiles is 1.03 sps, 95% CI [0.93, 1.17], and at 96 months, this difference is 0.82 sps, [0.71, 0.94]. Thus, the range of typical variability among children decreased a small amount (about 20%) over 5 years.

### Estimate the Distribution of Ages at Which Typically Developing Children Become Adultlike in Their Speech Rate Development

#### Baseline for Adultlike Rate

Adults who completed the task had articulation rates between 2.9 and 4.2 sps with a mean rate of 3.6. The 10th percentile for the adult rates was approximately 3.2 sps with 21 out of 23 speakers having an articulation rate faster than 3.2 sps. This rate was also the average articulation rate of the adult model in the recorded speech stimuli that the children heard on this task. Because most of the adult speakers surpassed this rate, we used 3.2 sps as a baseline for the lower range of typical variation in adult articulation rate.

#### Age of Crossing the Adultlike Baseline

We calculated the age at which each percentile line crossed the 10th percentile articulation rate for adults. The estimated articulation rate for the 50th percentile crossed the 3.2-sps baseline at age 75 months, 95% CI [71, 80]. The 75th and 90th percentiles crossed the same threshold approximately 2 and 3 years earlier, respectively, at 53 months, [50, 57], for the 75th percentile and at 41 months, [36, 44], for the 90th percentile. For the 25th percentile, the expected age of crossing the adult baseline was 99 months, [92, 110]. The difference in the expected ages for the 25th and 75th percentiles was 46 months, [37, 57]. In other words, it takes approximately 4 years for the middle 50% of children to reach adultlike performance; the range of typical articulation rates seems to develop slowly over the course of years, at least with respect to reaching the adultlike baseline of 3.2 sps. Finally, for the 5th and 10th percentiles, 90% and 55% of sampled percentile lines, respectively, did not cross the 3.2-sps baseline before 120 months of age. This result was expected given that 3.2 sps is based on the 10th percentile of the adult sample.

## Discussion

In this study, we provide the first large-sample developmental study of articulation rate, which is a measure of articulatory movement for speech (excluding pause time). Articulation rate is an indicator of motor ability and, because pauses are excluded, is thought to be less reflective of speaker-specific characteristics (Chon et al., 2012) than overall speech rate. Our sample included 570 typically developing children from early childhood (30 months, 2;6) to middle childhood (119 months, 9;11) who were performing an elicited sentence repetition task that systematically samples utterances between two and seven words in length. We identified and compared the joint effects of age and utterance length on articulation rate. We also created percentile growth curves to quantify the amount of typical variation in articulation rate over time. In addition, we collected an adult comparison sample so that we could characterize the range of performance on the same tasks for adults and estimate when children become adultlike in articulation rate. The results of the study provide the first comprehensive account of the development of articulation rate from early childhood into middle childhood. These results help us to recontextualize inconsistent findings from earlier small-scale studies. They also lay the groundwork for the study of overall speech rate (inclusive of pauses) and move us toward a comprehensive understanding of the interactions between articulatory movement for speech and pause behavior. Here, we discuss our results for each research objective and then as part of the larger body of research on articulation rate.

### Growth in Articulation Rate as a Function of Age and Utterance Length

Not surprisingly, results of this study showed that on average articulation rate of typically developing children increased with age from early childhood into middle childhood. The effect of age on articulation rate held within utterances of all lengths. For example, 7-year-olds had a faster articulation rate on average than 4-year-olds on two-word utterances, three-word utterances, and so on. Moreover, the degree of difference among ages was relatively modest, so that 7-year-olds were 7%–17% faster than 3-year-olds on average, depending on the utterance length. These age-related improvements in articulation rate would seem to be attributable to speech motor development. Nip and Green (2013), for example, highlighted the age range from 4 to 13 years as a phase of speech motor development during which articulator (lower lip) displacement decreases and articulation rate increases. That is, children may articulate more quickly by decreasing the amplitude of their movements. Goffman and Smith (1999), in contrast, observed smaller and slower (lower lip) movements in 4-year-olds versus 7-year-olds and no significant difference in movement durations between 7-year-olds and adults. In this case, children may articulate more quickly by moving the articulators at a higher velocity. This study did not examine articulatory kinematics, and therefore, we are unable to pinpoint the underlying source(s) of changes to articulation rate. However, given the variability among children—Vick et al. (2012), for example, identified three different profiles of articulatory behavior in typically developing preschoolers—it is likely that children may use different articulatory strategies to attain increases in articulation rate.

Our adult comparison sample provides an expected end point of articulation rate development (in the early 20s). Specifically, the college-age young adults we examined had an average overall articulation rate of 3.6 sps, and our 8- to 9-year-old speakers had an average overall rate of 3.4 sps: a 6% increase over the course of a 10+ year increase in age. It is not clear how this final increase in average articulation rate develops: as a protracted period of very slow maturation, as an increase followed by a plateau, or as plateau followed by later increase. Darling-White and Banks (2021), who examined articulation rate in sentence repetition on 10- to 14-year-olds using stimuli from the same task as this study, did not detect a significant effect of age. After the age of 10 years, articulation rate may grow at a small hard-to-detect rate, or it may plateau with a relatively sharp increase in the teenage years. Nip and Green (2013) provide support for the latter possibility by showing an increase in articulatory movement for speech between ages 13 and 16 years. Wong et al. (2011) also provide support for later rate development, finding a decrease in average syllable duration between ages 9 and 13 years (199 ms, *SE* = 6) and 14 and 18 years (177 ms, *SE* = 8).

In this study, we also found that children and adults used a faster average articulation rate in longer utterances. An additional word in utterance length predicted a corresponding increase of average articulation rate on the order of 0.05–0.15 sps, depending on the age of speaker. This effect matches the findings of Redford (2014), where the average articulation rate in sentence repetition for 5- to 7-year-olds increased by .05 sps for each additional syllable in utterance length. Our findings are also consistent with those of Darling-White and Banks (2021), who found that articulation rate increased with utterance length in 10- to 14-year-olds from approximately 3 sps for two-word utterances to approximately 4 sps for seven-word utterances. Our results showed a similar magnitude of effect with 8- to 9-year-old children having rate that increased from 2.9 sps for three-word utterances to 3.7 sps for seven-word utterances. Haselager et al. (1991) showed similar effects of age and length on articulation rate in conversational speech in Dutch children.

One interpretation for the utterance length effect on articulation rate is prosody. Crystal and House (1990) found that, for adult speakers, average syllable duration increased with the proportion of stressed syllables in an articulatory “run” (uninterrupted run of speech between two pauses). If longer utterances are more likely to have unstressed function words, then the proportion of “fast,” unstressed syllables should be larger in these utterances. We did not investigate how the composition of stressed vowels in utterances or in runs of speech affected articulation rate; thus, any conclusions about prosody in this study are speculative. However, this is clearly an area for future investigation.

Broadly speaking, our results confirm findings from earlier studies (rates increase with age and increase with utterance length), but importantly, the large-scale sample of this study helps put those findings on firmer grounding. Moreover, we were able to look at age and rate effects, finding that, on average, a 1-year increase in age was approximately the same as a one-word increase of utterance length in terms of effect size. These results provide an important foundation for understanding the impacts of utterance length on articulation rate. Future work will examine pause behavior along with articulation rate data and will integrate speech intelligibility findings for these children so that we can begin to understand how rate and intelligibility may interact with development. Ultimately, this information will be used to advance the creation of assessment tools, clinical decision-making rubrics, and intervention approaches to improve functional speaking ability for children with speech disorders.

### Overall Growth in Articulation Rate Between 2 and 9 Years

After we pooled articulation rate from different utterance lengths together, we estimated developmental percentiles for articulation rate. We found that the average articulation rate, and the major percentiles in Table 4, increased with age. The overall magnitude of the growth was on the order of 1 sps over the course of 7 years, starting from 2.6 sps at age 30 months (2;6) and ending at 3.5 sps at 114 months (9;6). Growth here was nonlinear, and Figure 3 does not show a straight line. Articulation rate first grows quickly and then it grows more slowly. For the 50th percentile, articulation rate increased a total of 0.5 sps from 30 to 60 months (2;6 to 5;0) and then 0.4 sps from 60 to 114 months (5;0 to 9;6). Growth from 30 to 60 months is slightly faster for the slower-speaking children in the 5th and 10th percentiles with a change of 0.6 sps. Conversely, growth is slightly slower for faster-speaking children in the 90th and 95th percentiles with a change of .4 sps. One explanation for this difference is that children in the higher percentiles begin with faster articulation rates and therefore do not have as far to advance before becoming adultlike. This same phenomenon was observed in our companion study of speech intelligibility (Hustad et al., 2021).

Given the effect of utterance length, our growth rate norms cannot be compared with other studies directly. Instead, we establish our comparisons on data reported for similar utterance lengths in elicitation tasks. In this context, our articulation rate data are consistent with results from prior studies of sentence repetition with known sentence lengths. Redford (2014) reported 5-, 6-, and 7-year-olds with rates of 3.3, 3.6, and 4 sps for utterances that were 8–9 syllables in length, making their results most comparable to our six- or seven-word sentences. The seven-word utterances used in our study had means of 3.3, 3.5, and 3.7 sps for 5-, 6-, and 7-year-old children, respectively. The 8- to 9-year-olds in this study had a seven-word rate of 3.7 ± 0.4 sps (*M* ± *SD*), and the adults varied at 4.0 ± 0.3. Darling-White and Banks (2021) showed similar values for 10- to 14-year-olds with rates between 3.9 ± 0.6 and 4.1 ± 0.7.

### Distribution of Ages at Which Typically Developing Children Become Adultlike

We estimated when typically developing children demonstrated an adultlike articulation rate by asking when the developmental growth curve percentiles crossed 3.2 sps. We chose this value because it reflects the lower end of expected adult performance (10th percentile). In addition, and coincidentally, it was the articulation rate of the elicitation stimuli presented to speakers during the task. Based on the growth curve summary in Table 4, our findings indicate that we expect 25% of typically developing children to reach 3.2 sps at 54 months (4;6), 50% at 72 months (6;0), 75% at 84 months (7;0), and 90% at 114 months (9;6). Some children in the higher-performance percentiles will show adultlike articulation rate earlier than these thresholds, but it takes years for most children to reach this rate. This time scale is in line with a protracted time course of speech motor development, as noted earlier.

### Variability Within and Between Speakers

A crucial result of this study is that variability in articulation rate was larger *within* ages than *between* ages. An example is the difference between 90th and 10th percentiles. Within a given age, this difference was between 0.5 and 0.8 sps. However, the same difference of 0.8 sps can also be observed within the 10th percentile between children at 36 months (3;0) and children at 96 months (8;0). That is, the change *within* an age of 80-percentile points is equivalent to about 5 years of change *between* ages. This high level of variability was also present in our adult speakers (with an sps range of 1.3). This variability was observed in a highly controlled task where speakers all heard and repeated the same stimuli, which were systematically controlled for utterance length. Studies with spontaneous speech, repetition of live models, or other contingent procedures would likely show even more variability. Similarly, interactive procedures might yield even more variability: For Logan et al. (2011), the examiners spoke approximately 0.4 sps faster with older (8- to 11-year-olds) children than younger children (5- to 8-year-olds) on a sentence modeling task and on a structured conversation task. It is also useful to note that variability in articulation rate (within utterances) is a desirable feature for natural speech (Eefting, 1988; cited in Crystal & House, 1990), contributing to prosodic variation and reducing the perception of monotony.

Considering the large degree of variability within ages and slow growth between ages based (to our knowledge) on the largest sample of typically developing children studied to date, we can re-interpret some previous findings in the literature. Because it can take years for statistically clear age-related group differences to emerge, long-range age differences should be more reliable than short-range differences. For example, Nip and Green (2013) found significant group differences in articulation rate on sentence repetition when comparing 13-year-olds with 4-, 7-, and 10-year-olds; 16-year-olds with 4-year-olds; and adults with 4- and 7-year-olds, but *not* among the 4-, 7-, and 10-year-olds.

Some inconsistent findings in the literature may, in fact, be reflective of cases where variability in rate obscured age effects. For example, in a cross-sectional study, Walker et al. (1992) measured articulation rate in different speaking conditions. They found a 0.3-sps increase in average articulation rate from 3.6 sps in twenty 3-year-olds to 3.9 sps in twenty 5-year-olds. Using the same procedures, Walker and Archibald (2006) measured articulation rate longitudinally in a sample of 16 children. They found average articulation rates of 3.4, 3.15, and 3.3 sps at ages 4, 5, and 6 years old, respectively. Both results are consistent with children's articulation rate being highly variable and sensitive to small sample sizes, so that small year-over-year changes are difficult to detect.

Our findings were to an extent foreshadowed by Pindzola et al. (1989). After not finding a clear developmental increase in articulation rate in 3- to 5-year-olds, they speculate that “large variation in rates, seen in the present study, probably obscures any age-related trends, especially when so few subjects were used. Interpretations of developmental trends also are hampered in that only three very similar age groups were studied.” This study examined a large number of children, stratified for age and covering a broad age range on a controlled repetition task. In this context, we were able to detect clear developmental trends, spanning a continuum of higher- and lower-performing children at each age. Our results, in light of prior findings, highlight the need for large-scale studies on rate in other speaking contexts (spontaneous and conversational speech samples).

### Limitations and Future Directions

A key limitation of this work was that the speech task followed an utterance-level, picture-prompted repetition procedure, so the articulation rates were from repeated speech, as opposed to other production contexts such as spontaneous speech, conversational speech, or recited/read narration. Although speakers were not instructed to match the rate of the stimuli, and indeed most adults and older children exceeded the model speech, it was conceivable that the model speech influenced the rate used by the children. The prerecorded utterances of Redford (2014), for example, had an average rate of 4.0 sps (compared with 3.2 sps here), and two of the age groups in that study showed faster mean articulation rates than their counterparts in this study. Future studies should examine articulation rate development in different speaking tasks, particularly spontaneous speech, to determine the extent to which findings from a more structured task such as the one used in this study generalize to speech produced in less-structured contexts.

Using sentence repetition afforded greater experimental control as children did not have to formulate utterances in real time during speaking. However, even repeated speech proved taxing for younger children on longer utterances. Future work should expand the study of rate to include measurement of pauses (speaking rate) and pause probability as functions of age and utterance length. Darling-White and Banks (2021) identified a developmental ceiling on these effects as time spent pausing was not related to age in 10- to 14-year-olds. However, similar effects in younger children have not been examined.

In addition to minimizing cognitive or pragmatic demands, our sentence elicitation task also reduced motor demands by only requiring the child to speak comfortably. Articulation rate can also be measured in maximum performance tasks, and data from such tasks could reveal more extreme developmental patterns and clarify how articulatory capacity changes with age, albeit at the expense of naturalness. Nip and Green (2013), for instance, found a significant difference between 4-year-olds and 7-year-olds on rate of speeded repetition of the syllable “buh” (but not for comfortable sentence repetition).

Given the age expectations for articulation rate established in this study, one important follow-up question is whether articulation rate can differentiate children with dysarthria or other speech disorders from those on the margins of typical development. Future work from our group will examine how children with cerebral palsy (with and without speech motor impairment) speak with respect to age percentiles and use receiver operating characteristic curves to measure the sensitivity and specificity of different articulation rate benchmarks.

Another important set of questions relates to the impact of rate development on speech intelligibility. A companion paper from our group established parallel growth curves for speech intelligibility development (Hustad et al., 2021), but we have not yet examined rate characteristics of the most and least intelligible children by age. This information could have important implications for our understanding of functional speech development as well as to help guide identification of children with speech disorders.

### Clinical Implications

There are several important clinical implications from this study that may have some relevance to clinical practice. Articulation rate develops quickly until the age of 5 years after which point it settles into a more gradual growth rate. Our results suggest some developmental milestones that may be useful benchmarks for clinicians to consider when assessing articulation rate in children suspected of having speech disorders. Children should have an articulation rate of at least 2.5 sps on repeated sentences by 4 years of age and at least 3 sps by around 10 years of age. These values reflect the 5th percentile of typical development. Given the broad range of typical developmental variability from which these benchmarks emerged, it is likely that children who fall well below these values may have an underlying disorder. While these results, in their current form, do not directly impact treatment, they do advance our understanding of how different facets of speech develop in children. In combination with our other companion studies, results advance our knowledge of straightforward and clinically interpretable measures that may ultimately inform differential diagnosis, particularly for children who are on the borderline of typical development. Further work is required to calibrate these milestones against speaking rate (inclusive of pauses) and against speakers with dysarthria and other speech disorders in order to identify diagnostic values.

## Footnote

1 There was one additional utterance used for task familiarization that we included in this study, for a total of 11 utterances of three words in length.
